# Effectiveness of the *SmartShield* for knowledge on sexual abuse prevention among primary school children in Malaysia: A quasi-experimental study

**DOI:** 10.1371/journal.pone.0334401

**Published:** 2025-10-09

**Authors:** Mohamad Najib Mat Pa, Mohd Noor Norhayati, Zaharah Sulaiman, Nik Hazlina Nik Hussain, Azizah Othman, Azliana Aziz

**Affiliations:** 1 Department of Medical Education, School of Medical Sciences, Universiti Sains Malaysia, Kubang Kerian, Kelantan, Malaysia; 2 Department of Family Medicine, School of Medical Sciences, Universiti Sains Malaysia, Kubang Kerian, Kelantan, Malaysia; 3 Women’s Health Development Unit, School of Medical Sciences, Universiti Sains Malaysia, Kubang Kerian, Kelantan, Malaysia; 4 Department of Paediatrics, School of Medical Sciences, Universiti Sains Malaysia, Kubang Kerian, Kelantan, Malaysia; 5 Department of Otorhinolaryngology-Head & Neck Surgery, School of Medical Sciences, Universiti Sains Malaysia, Kubang Kerian, Kelantan, Malaysia; Università degli Studi di Torino: Universita degli Studi di Torino, ITALY

## Abstract

Child sexual abuse is a significant public health issue with long-term psychological, physical, and social consequences. Despite increasing awareness, its prevention remains underrepresented in the Malaysian primary school curriculum. The *SmartShield* program was developed as a culturally relevant, age-appropriate, and video-based intervention to improve sexual education knowledge among primary school children. A quasi-experimental controlled trial was conducted using stratified multistage cluster sampling. Six government primary schools were randomly selected from five regions. In each school, one class from the lower and one from the upper primary were chosen. The sample size was estimated by comparing two means. The *SmartShield* intervention consisted of two modules per age group and was delivered through 20-minute video-guided sessions. Knowledge was assessed at baseline, post-Module 1 (Week 2), and post-Module 2 (Week 4) using validated questionnaires. Data were analyzed using repeated measures ANOVA, adjusting for sex and region. A total of 881 children for each lower and upper primary student were recruited. The intervention group showed significantly greater improvements in knowledge scores compared to the control group across all modules and time points (p < .001). Among lower primary students, adjusted mean scores increased from 78.5% to 97.1% in the intervention group versus 80.1% to 90.3% in the control group. Among upper primary students, knowledge scores rose from 79.5% to 97.8% in the intervention group, compared to 77.6% to 95.1% in the control group. The *SmartShield* program was effective in enhancing child sexual abuse knowledge among Malaysian primary school children.

## Introduction

Child sexual abuse (CSA) is a widespread public health concern that has long-lasting consequences on children’s physical, mental, and developmental well-being. The World Health Organization (WHO) defines CSA as involving a child in sexual activities that they do not fully understand, cannot consent to, or are not developmentally ready for, and that violate legal or societal norms [1]. CSA includes non-contact acts, physical contact, and penetrative abuse [2]. Globally, it is estimated that 7–37% of women and 5–10% of men have experienced some form of CSA during childhood. However, the true prevalence is likely higher due to widespread underreporting associated with stigma, fear, and cultural taboos [3].

Victims of CSA face not only significant mental health challenges, such as depression, anxiety, substance addiction, and suicidal ideation, but they are also likely to develop long-term physical health conditions, including diabetes, obesity, and coronary heart disease [4]. Furthermore, CSA can severely disrupt psychosocial development and academic achievement [5]. In Southeast Asia, CSA continues to be a pressing issue. Alarmingly high prevalence rates have been reported in the region, with 41.6% of children in Vietnam and 22.4% in Malaysia affected [6]. These figures underscore the urgent need for comprehensive preventive interventions that can be implemented early in a child’s life.

Empowering children with knowledge about body safety, personal boundaries, and appropriate responses to potential threats is critical for effective CSA prevention. School-based sexual abuse prevention programs have been shown to improve primary school children’s knowledge, especially when grounded in behavioral theories and tailored to their developmental stage [7]. Despite these benefits, many children still lack accurate knowledge about sexual health and personal safety. For example, a study in Spain found that adolescents with lower levels of formal education often held misconceptions and lacked understanding about sexuality [8].

In Malaysia, efforts have been made to integrate sexuality education into the school curriculum through culturally sensitive approaches. However, CSA prevention remains a relatively neglected component [9]. There is a clear need for structured, culturally relevant, and developmentally appropriate programs designed to address CSA in the Malaysian context. The *SmartShield* educational program was developed as an innovative, video-based teaching intervention to increase CSA prevention knowledge among Malaysian primary school children. The module combines culturally relevant content, interactive learning strategies, and child-centered pedagogy, and is tailored for lower and upper primary school students. While the *SmartShield* program appears promising as a preventive tool, its effect on children’s understanding of CSA has not yet been thoroughly assessed. Therefore, this study aimed to determine the effectiveness of the *SmartShield* program in improving child sexual abuse prevention knowledge among lower and upper primary school students in Malaysia.

## Materials and methods

This study employed a quasi-experimental controlled trial study design. The target population comprised students from lower (ages 7–9) and upper (ages 10–12) primary levels enrolled in government schools. These age groups were selected to represent the corresponding stages of primary education. Children with diagnosed learning disorders (e.g., attention deficit disorder, dyslexia), as reported by teachers or school health records, and those absent during intervention sessions were excluded. The inclusion and exclusion criteria were applied to intervention and control groups to minimize selection bias.

A stratified multistage cluster sampling method was used. The state of Malaysia is divided into five regions, namely: Northern, Middle, Southern, Eastern, and East Malaysia regions. Within each region, six government primary schools were randomly selected using lists obtained from respective State Education Departments. From each school, one class from the lower primary and one from the upper primary were randomly chosen. All students in the selected classes were invited to participate in the study.

The required sample size was calculated using Power and Sample Size Calculation software (Microsoft Corp., 2012) based on the comparing two means. Using a significance level of 0.05, a power of 80%, and a standard deviation (SD) of 3.47 derived from a New Zealand CSA prevention study (‘Feeling Special, Feeling Safe’), the expected minimum detectable difference was set at 2 [10]. The control-to-intervention group ratio was fixed at 1:1. After adjusting for a design effect of 2, the minimum required sample size was 380 children. However, to account for a 20% non-response rate, the final calculated sample size was 456 children per group for each lower and upper primary school.

The intervention comprised two educational programs, *SmartShield* 1 (for lower primary) and *SmartShield* 2 (for upper primary). These programs featured age-appropriate content tailored for primary school children and were aligned with the Malaysian Ministry of Education curriculum. *SmartShield* 1 focused on body awareness, personal boundaries, types of touch (safe, unsafe, uncomfortable), and self-protection strategies. It emphasized body privacy, recognizing inappropriate touch, and appropriate responses in unsafe situations. The content of *SmartShield* 1 was divided equally into Module 1A and Module 1B.

*SmartShield* 2 addressed puberty, reproductive health, sexual grooming, personal safety, and social responsibility. It aimed to enhance students’ understanding of physiological changes, reproductive knowledge, and the importance of personal and social boundaries. The content of *SmartShield* 2 is equally divided into Module 2A and Module 2B. Local cultural expectations guided the development of *SmartShield*. Careful attention was given to the choice of words, the style of illustrations, and the way sensitive topics were introduced. These considerations made the module acceptable and meaningful for Malaysian children, while also allowing space for adaptation in other cultural settings.

The *SmartShield* 1 and *SmartShield* 2 questionnaires were developed to assess knowledge related to child sexual abuse among lower and upper primary school students, respectively. The *SmartShield* 1 questionnaire covers questions related to the topics in Module 1A and Module 1B. It consists of 21 items with an internal consistency of 0.692 [11]. The *SmartShield* 2 questionnaire covers a broader range of topics related to Module 2A and Module 2B. It consists of 47 items and an internal consistency was 0.838 [11].

Knowledge scales were rated on a 3-point scale: 2 for a correct answer, 1 for unsure, and 0 for a wrong answer. Scores for negatively worded items were reversed. The *SmartShield* 1 questionnaire had a minimum score of 0 and a maximum score of 42, while the *SmartShield* 2 questionnaire had a minimum score of 0 and a maximum score of 94. Raw scores were summed and converted into percentage scores. The higher the scores, the more knowledgeable the students are.

Data collection was conducted over a four-week period. Informed consent was distributed to the parents or guardians through the teachers. It was informed that their participation in research is voluntary, and they are permitted to withdraw. The respondents who agreed to participate in the research responded to the consent form and completed the self-administered questionnaire. The recruitment period for this study was from 7 June 2023–28 December 2023 and from 1 March 2024–27 August 2024. At week 0, lower primary students completed a sociodemographic form followed by the baseline *SmartShield* 1 questionnaire. Immediately thereafter, the intervention group received *SmartShield* Module 1A, while the control group continued with the standard national curriculum without exposure to the intervention. At Week 2, both groups completed the *SmartShield* 1 questionnaire related to Module 1A. Subsequently, the intervention group received *SmartShield* Module 1B. At Week 4, both groups completed the *SmartShield* 1 questionnaire related to Module 1B.

All assessments were administered during regular school hours under the supervision of classroom teachers. For the lower primary students, the intervention was delivered in two separate 20-minute sessions (Modules 1A and 1B) to optimize attention span and learner engagement. Each session involved interactive video content facilitated by classroom teachers in the school setting. The control group did not receive any component of the *SmartShield* modules throughout the study period and continued with the standard curriculum. The same data collection procedures were applied to upper primary students using the *SmartShield 2* modules (2A and 2B) and *SmartShield* 2-related questionnaires.

Data was analyzed using IBM SPSS Statistics version 29.0 (SPSS Inc., 2019). Initial data cleaning was performed to ensure accuracy. A repeated measures analysis of variance (RM-ANOVA) was conducted to assess within- and between-group differences in knowledge scores over time. Before running the RM-ANOVA, the standard assumptions were examined. The data satisfied the requirements for normality, homogeneity of variances, and sphericity, providing confidence that the results were interpreted appropriately. Adjustments were made for potential confounders, including sex and geographical zone.

This study obtained ethical approvals from the Human Research Ethics Committee, Universiti Sains Malaysia (USM/JEPeM/20110554) and the Ministry of Education Malaysia (KPM.600-3/2/3-eras-16567). Written informed consents were obtained from parents or guardians.

## Results

### *SmartShield* 1 for lower primary school children

Participants for the intervention and control groups were recruited, with 96.5% (440/456) and 96.7% (441/456) response rates, respectively. The sociodemographic characteristics of the participants in the lower primary school children are shown in Table 1.

**Table 1:** Sociodemographic characteristics of lower primary school children.

Variable	Intervention (n = 440)		Control (n = 441)		*p* value^a^
	n	(%)	n	(%)	
Sex					
Male	212	(48.2)	228	(51.7)	.944
Female	228	(51.8)	213	(48.3)	
Zone					
Eastern	88	(20.0)	87	(19.7)	.844
Southern	89	(20.2)	88	(20.0)	
Northern	88	(20.0)	87	(19.7)	
Middle	89	(20.2)	89	(20.2)	
East Malaysia	86	(19.5)	90	(20.4)	
Race					
Malay	422	(95.9)	416	(94.3)	.030
Non-Malay	18	(4.1)	25	(5.7)	

^a^ Chi-Square.

The group-time interaction of the RM-ANOVA showed a significant difference in mean knowledge score changes between the intervention and control groups after adjusting for sex and zone for Module 1A (p < .001), Module 1B (p < .001), and overall, for the combination of Module 1A and Module 1B (p < .001) (Table 2).

**Table 2:** Comparisons of knowledge on sexual education among lower primary school children between intervention and control groups.

Group	n	Desc Mean ^a^ (SD ^b^)		EMM ^c^ (95% CI ^d^)		F stat ^e^ (d ^f^)	*p* value ^g^
		Base ^h^	Post ^i^	Base ^h^	Post ^i^		
Module 1A							
Intervention	440	85.1 (9.57)	98.3 (4.07)	85.1 (84.2, 86.0)	98.3 (97.6, 99.0)	49.35 (1)	<.001
Control	441	85.6 (9.84)	92.0 (9.77)	85.6 (84.7, 86.5)	92.0 (91.3, 92.7)		
Module 1B							
Intervention	440	71.9 (13.97)	95.9 (5.35)	71.9 (70.6, 73.2)	95.9 (94.9, 96.9)	14.80 (1)	<.001
Control	441	74.7 (14.20)	88.6 (14.75)	74.7 (73.4, 76.0)	88.6 (87.6, 89.6)		
Overall							
Intervention	440	78.5 (9.64)	97.1 (3.29)	78.5 (77.6, 79.4)	97.1 (96.4, 97.8)	43.50 (1)	<.001
Control	441	80.1 (9.93)	90.3 (9.54)	80.1 (79.2, 81.1)	90.3 (89.6, 91.0)		

^a^Descriptive mean.

^b^Standard deviation.

^c^Estimated marginal mean.

^d^Confidence interval.

^e^F statistic.

^f^Degree of freedom.

^g^Group-time interaction of repeated measure analysis of variance after adjusting for sex and zone.

^h^Baseline.

^i^Post-intervention.

The group effect of the RM-ANOVA showed a significant difference in mean knowledge score changes between the intervention and control groups after adjusting for sex and zone for Module 1A [mean (95% CI): 91.7 (91.1, 92.3) versus 88.8 (88.2, 89.4), p < .001], Module 1B [mean (95% CI): 83.9 (83.1, 84.7) versus 81.6 (80.8, 82.5), p < .001] and overall, for combination of Module 1A and Module 1B [mean (95% CI): 87.8 (87.3, 88.4) versus 85.2 (84.7, 85.8), p < .001].

### *SmartShield* 2 for upper primary school children

Participants for the intervention and control groups were recruited, with 441 and 436 participants, respectively. The response rates were 96.7% (441/456) for the intervention group and 95.6% (436/456) for the control group (Table 3).

**Table 3:** Sociodemographic characteristics of upper primary school children.

Variable	Intervention (n = 441)		Control (n = 436)		*p* value^a^
	n	(%)	n	(%)	
Sex					
Male	209	(47.4)	223	(51.1)	.421
Female	232	(52.6)	213	(48.3)	
Zone					
Eastern	89	(20.2)	87	(20.0)	.799
Southern	87	(19.7)	89	(20.4)	
Northern	88	(20.0)	83	(19.0)	
Middle	88	(20.0)	87	(20.0)	
East Malaysia	89	(20.2)	90	(20.6)	
Race					
Malay	401	(90.9)	415	(95.2)	<.001
Non-Malay	40	(9.1)	21	(4.8)	

^a^Chi-Square.

The group-time interaction of the RM-ANOVA showed a significant difference in mean knowledge score changes between the intervention and control groups after adjusting for sex and zone for Module 2A (p < .001), Module 2B (p < .001) and overall, for combination of Module 2A and Module 2B (p < .001) (Table 4).

**Table 4:** Comparisons of knowledge for sexual education among upper primary school children between intervention and control groups.

Group	n	Desc Mean ^a^ (SD ^b^)		EMM ^c^ (95% CI ^d^)		F stat ^e^ (d ^f^)	*p* value^g^
		Base ^h^	Post ^i^	Base ^h^	Post ^i^		
Module 2A							
Intervention	441	86.3 (8.90)	98.8 (2.60)	86.3 (85.4, 87.1)	98.8 (98.4, 99.1)	25.85 (1)	<.001
Control	436	83.8 (9.39)	92.0 (9.77)	83.8 (83.0, 84.7)	97.7 (97.3, 98.1)		
Module 2B							
Intervention	441	74.2 (9.53)	95.9 (6.26)	74.2 (73.2, 75.1)	95.9 (95.1, 96.7)	62.50 (1)	<.001
Control	436	72.5 (10.33)	90.3 (10.47)	72.5 (71.6, 73.5)	90.3 (89.5, 91.1)		
***Overall***							
Intervention	441	79.5 (7.44)	97.8 (2.71)	79.5 (78.8, 80.2)	97.8 (97.5, 98.2)	53.71 (1)	<.001
Control	436	77.6 (7.98)	95.1 (5.00)	77.5 (76.8, 78.3)	95.1 (94.8, 95.5)		

^a^Descriptive mean.

^b^Standard deviation.

^c^Estimated marginal mean.

^d^Confidence interval.

^e^F statistic.

^f^Degree of freedom.

^g^Group-time interaction of repeated measure analysis of variance after adjusting for sex and zone.

^h^Baseline.

^i^Post-intervention.

The group effect of the RM-ANOVA showed a significant difference in mean knowledge score changes between the intervention and control groups after adjusting for sex and zone for Module 2A [mean (95% CI): 92.5 (92.0, 93.0) versus 90.8 (90.3, 91.2), Module 2B [mean (95% CI): 85.0 (84.4, 85.7) versus 81.4 (80.8, 82.1), p < .001], and overall, for combination of Module 2A and Module 2B [mean (95% CI): 88.6 (88.2, 89.1) versus 86.4 (86.0, 86.8), p < .001].

## Discussion

This study provides evidence for the effectiveness of the *SmartShield* sexual abuse prevention program in enhancing knowledge among Malaysian primary school children. The findings demonstrated significant improvements in knowledge for both lower and upper primary students in the intervention group compared to their peers in the control group. These findings support the growing body of literature that affirms the importance of school-based, age-appropriate interventions in improving children’s awareness of sexual abuse and self-protection strategies.

International evidence supports the effectiveness of school-based CSA prevention programs in enhancing knowledge and protective behaviors among children. The IGEL program in Germany, developed for third-grade students, demonstrated significant improvements in CSA-related knowledge. The IGEL, meaning ‘hedgehog’ in German, symbolizes the capacity for self-protection [12]. Similarly, the Child Sexual Abuse Prevention Education using Hybrid Application (CSAPE-H) in South Korea showed positive outcomes through a blended learning approach [13]. The ESPACE program (Éducation, Sensibilisation, Prévention, Alerte, Conseil, Écoute), originally developed in Canada and adapted for Francophone contexts, also reported substantial gains in children’s awareness and resistance strategies [14]. These findings are consistent with the outcomes of this study and reinforce the notion that well-structured, context-sensitive prevention education can play a significant role in child protection. However, unlike many interventions tested in high-income countries, *SmartShield* offers a unique contribution from a low- to middle-income context in Southeast Asia, with content adapted to the Malaysian cultural and educational setting.

While previous studies in Malaysia have primarily focused on general reproductive health or moral education [15,16], the *SmartShield* modules specifically target CSA prevention, making them novel and contextually relevant. The digital, video-based delivery format allowed for consistent messaging, ease of implementation, and scalability, especially in resource-constrained environments [17,18]. Short, focused sessions align well with students’ attention spans and can be feasibly integrated into existing school timetables without disrupting core academic instruction.

Cultural relevance was a key consideration in the development of the *SmartShield* modules [19]. The use of familiar settings, characters, and language aligned with the local context, which supported student engagement and understanding. These culturally grounded elements helped make the content more accessible and meaningful. Ensuring cultural adaptability is essential to increase the effectiveness of CSA prevention programs across diverse populations [20].

The *SmartShield* program was designed to be led by teachers, making them easier to implement within existing school systems. Programs delivered by familiar educators are more effective, as children feel more comfortable and engaged [21]. This teacher-led approach also supports cost-effectiveness and long-term sustainability, especially in resource-limited settings [22]. Evidence from programs like the Doll Program in China further demonstrates that teacher-facilitated delivery enables consistent learning outcomes and wider program reach [20]. The digital and interactive nature of *SmartShield* is also consistent with findings from other technology-enhanced programs, such as the Sexual Abuse Prevention Mobile Application (SAP_MobAPP) in Korea which showed that digital tools can effectively engage children and support knowledge retention without causing anxiety or distress [23].

While the present study focused on knowledge outcomes, it is essential to acknowledge that increased awareness and understanding are critical first steps toward behavior change [24]. Existing literature shows that well-structured CSA prevention programs can enhance children’s self-efficacy and promote protective behaviors, as demonstrated by gains in knowledge and self-protective actions among South Korean fifth graders [25] and improvements in protective skills and disclosure behavior among Iranian preschoolers [26]. A recent meta-analysis found that increasing knowledge is essential for changing behavior and suggested using combined behavioral strategies to improve program outcomes [27]. Although behavioral outcomes were not measured in the current study, the scenario-based structure of the *SmartShield* program may support applied learning and skill development. When paired with reinforcement strategies such as booster sessions or teacher-guided discussions, this approach holds strong potential to contribute to meaningful behavior change over time.

The findings also align closely with the goals of the United Nations Sustainable Development Goals (SDGs), particularly SDG 3 (Good health and well-being), SDG 4 (Quality education), and SDG 16 (Peace, Justice and Strong Institutions). By equipping children with knowledge and self-protection skills, *SmartShield* contributes to improving child health outcomes and promoting safe learning environments. These objectives are in line with the United Nation’s 2030 Agenda, which emphasizes the importance of ensuring healthy lives, inclusive education, and protection from all forms of violence [28].

The *SmartShield* program could be a good fit for the national school curriculum. Including it in regular teaching, along with proper teacher training and follow-up, can help improve child protection across the country. Primary school teachers in China supported CSA prevention; they were more likely to use the programs well if they had received training [29]. Giving teachers the right support can help make the program work better, keep children safe, and support safer school communities.

This study has a few limitations. Although the quasi-experimental design is suitable for real school settings, it does not establish cause and effect. As a result, it is uncertain whether the program directly caused the outcomes because other factors, such as teacher enthusiasm, prior exposure to similar content, classroom environment, or student motivation, may have influenced the results. The study relied on self-reported questionnaires, which may have led to biased responses. In addition, there was no long-term follow-up to assess whether the knowledge gained was retained over time.

Future research should examine the long-term impact of the *SmartShield* program by following up with participants over an extended period. This would help determine whether the knowledge gained through the program is maintained over time and whether it leads to meaningful behavioral changes. Simple steps such as short refresher activities during the school year can help children keep the key messages alive in their memory. Elements of *SmartShield* could also be woven into everyday co-curricular activities, so that protective skills become part of normal school life. Involving parents and caregivers adds another layer of support, creating opportunities for children to practice what they have learned at home and strengthening their ability to use these skills in real situations. It is also important to assess if these changes result in improved self-protection skills in real-life situations. Such evidence would provide a stronger understanding of the program’s effectiveness beyond short-term learning outcomes.

## Conclusion

This study demonstrated that the *SmartShield* sexual abuse prevention program was effective in significantly enhancing knowledge related to child sexual abuse prevention among both lower and upper primary school children in Malaysia. Age-appropriate, culturally contextualized, and video-based educational interventions can be successfully integrated into school settings to improve children’s understanding of body safety, personal boundaries, and protection strategies. Given the substantial knowledge gains observed in the intervention group compared to controls, the *SmartShield* program holds substantial promise as a scalable component of Malaysia’s national child protection strategy.

## Supporting information

S1 Data Knowledge Level 1. (XLS)

S2 Data Knowledge Level 2. (XLS)
